# Diapause characterization in the invasive alien mosquito species *Aedes koreicus*: a laboratory experiment

**DOI:** 10.1186/s13071-022-05376-7

**Published:** 2022-09-06

**Authors:** Giovanni Marini, Daniele Arnoldi, Enrico Inama, Annapaola Rizzoli

**Affiliations:** Research and Innovation Centre, Fondazione Edmund Mach, San Michele All’Adige, TN Italy

**Keywords:** Egg, Diapause, Hatching, Overwintering, *Aedes koreicus*, Mosquito bionomics, Invasive species

## Abstract

**Abstract:**

*Aedes koreicus* is an invasive alien mosquito species native to Asia now introduced in several European countries, including northern Italy. In this temperate region, mosquito populations survive cold winter temperatures thanks to diapausing eggs or adults, depending on the species. In its native area, *Ae. koreicus* was reported to overwinter in the egg stage, but to the best of our knowledge, it is not confirmed whether overwintering eggs are actually diapausing or only in a quiescence stage, i.e., they might hatch as soon as external conditions are favorable. Based on previous laboratory studies, we established a diapausing *Ae. koreicus* colony, maintained at 21 °C with a photoperiod of 12L:12D. Females were allowed to lay eggs, which were consequently placed in water at different time intervals after oviposition, from 30 days to 5 months. We found that diapausing eggs younger than 3 months have a poor hatching rate, while after about 100 days we observed that almost all eggs hatched. Our findings highlight that water immersion alone did not lead to the hatching of eggs, as age was found to be a significantly important factor. We thus confirm effective diapause, occurring at the egg stage, for *Ae. koreicus* in a recently invaded area. Moreover, our quantification of diapause duration and hatching success might help in better designing future experiments and improving modeling efforts.

**Graphical Abstract:**

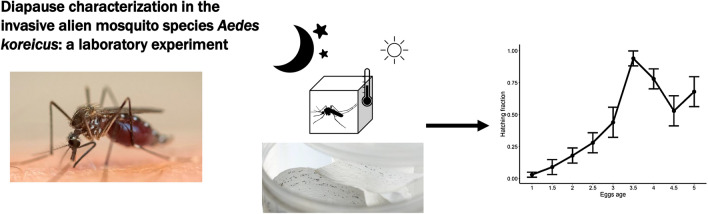

*Aedes koreicus* (Edwards, 1917) is an invasive alien mosquito species native to Asia [1]. It was recorded for the first time outside its native range in Europe (Belgium) in 2008 [2], and to date it has successfully established in several European countries [3]. In particular, in Northern Italy this mosquito species has been spreading quickly since its first detection in 2011 [4–8], probably thanks to its ability to tolerate the climatic conditions of the area [6].

This mosquito species has been shown experimentally to be competent to transmit the dog heartworm *Dirofilaria immitis* [9] and the chikungunya and Zika viruses [10, 11]. Despite its potential threat for both human and animal health, the biology and ecology of *Ae. koreicus* within areas of new invasion are still poorly known.

Winters in temperate areas might substantially constrain mosquito survival and development [12]. To face this challenge, mosquitoes have evolved a programmed dormancy known as diapause [12]. In particular, in its native area, *Ae. koreicus* has been reported to pass winter in the egg stage, hatching in the spring when the ice melts [13]. However, to the best of our knowledge, it is not confirmed whether overwintering eggs are actually diapausing or only in a quiescence stage. Diapause is defined as a form of dormancy that is hormonally programmed in advance of its onset and is not immediately terminated in response to favorable conditions. Such advanced programming distinguishes diapause from quiescence, a dormancy that is prompted in direct response to unfavorable environmental conditions and is immediately terminated upon the return of favorable factors. Hence, diapausing eggs respond to favorable external stimuli only after a fixed interval has elapsed [12].

We previously quantified how different constant temperatures can affect life history traits of this species by successfully establishing an *Ae. koreicus* laboratory colony [6]. Based on this previous investigation, we carried out additional experiments to confirm diapause in this mosquito species and evaluate its duration.

From the above-mentioned colony, we established a diapausing secondary colony. Larvae hatched from non-diapausing eggs from the primary colony were reared following the same protocol described in [6] but with a different temperature (21 °C) and photoperiod (12L:12D instead of 16L:8D) with 1 h of dawn and 1 h of dusk. This photoperiod simulates the day/night cycle occurring during the end of September in northern Italy. As observed in [14], a photoperiod of 11L:13D (mid-October) induces a 100% rate of diapausing eggs in *Ae. japonicus japonicus* (Theobald, 1901). We chose a slightly different photoperiod as we observed better fitness in our colony.

Females of the diapausing colony were allowed to lay eggs on a filter paper within an ovitrap filled with dechlorinated water and grass infusion. Strips with eggs were then removed once a week and placed in plastic cups partially covered with a lid to maintain high relative humidity. Eggs were maintained under the same conditions as the diapausing colony until the beginning of experiments.

Starting 1 month from oviposition, a subset of eggs laid at different dates were tested to evaluate whether diapause had been correctly induced by the rearing conditions described above. Eggs were divided into groups ranging from 5 to 26 eggs and three to twelve replicates were made for each tested age, namely 1, 1.5, 2, 2.5, 3, 3.5, 4, 4.5, and 5 months (30–150 days) after oviposition. Under a stereomicroscope, eggs were evaluated to avoid the presence of damaged, desiccated, or hatched eggs before forming the groups of eggs. Eggs were placed in plastic glasses filled with 100 ml of dechlorinated water. Four mg of finely ground cat food was added to the water as a hatching stimulus. The strips with eggs were left in water for 5 days and then left to dry for 2 days. Emerged larvae were counted during the flooding period and then removed. This procedure was carried out twice. At the end of this process, unhatched eggs were submerged in a commercial bleach solution (3.5% chlorine concentration) until the complete clarification of the egg shells. Under a stereomicroscope, eggs were examined and considered embryonated when complete segmentation, eye spots, and egg buster were observed. All the hatching experiments were performed under constant temperature (21 ± 1 °C) and relative humidity (75 ± 5%) with a photoperiod of 16L:8D with 1 h of dawn and 1 h of dusk.

Observed hatching rates are reported in Table 1 and Fig. 1. There is a clear increasing trend, implying a positive relationship between egg age and successful hatching. Such association is confirmed by a generalized linear model (GLM) with binomial-distributed error (estimated coefficients presented in Table 2) considering the fraction of hatched eggs and age (in months) as dependent and explanatory variables respectively. By looking at the number of embryonated eggs (obtained by summing the number of hatched eggs and the number of embryos found after bleaching) we can conclude that age seems to be a key factor determining hatching success.

**Table 1:** Initial number of eggs, number of embryonated eggs, and hatching rates (as fraction), with standard error, for the different tested ages

Age (months)	Initial eggs	Embryonated eggs	Hatching rate (with standard error)
1	238	185	0.03 ± 0.01
1.5	129	121	0.09 ± 0.03
2	121	115	0.18 ± 0.03
2.5	119	115	0.28 ± 0.04
3	62	54	0.44 ± 0.06
3.5	71	68	0.94 ± 0.03
4	92	83	0.78 ± 0.04
4.5	77	66	0.53 ± 0.06
5	59	51	0.68 ± 0.06

**Fig. 1:**
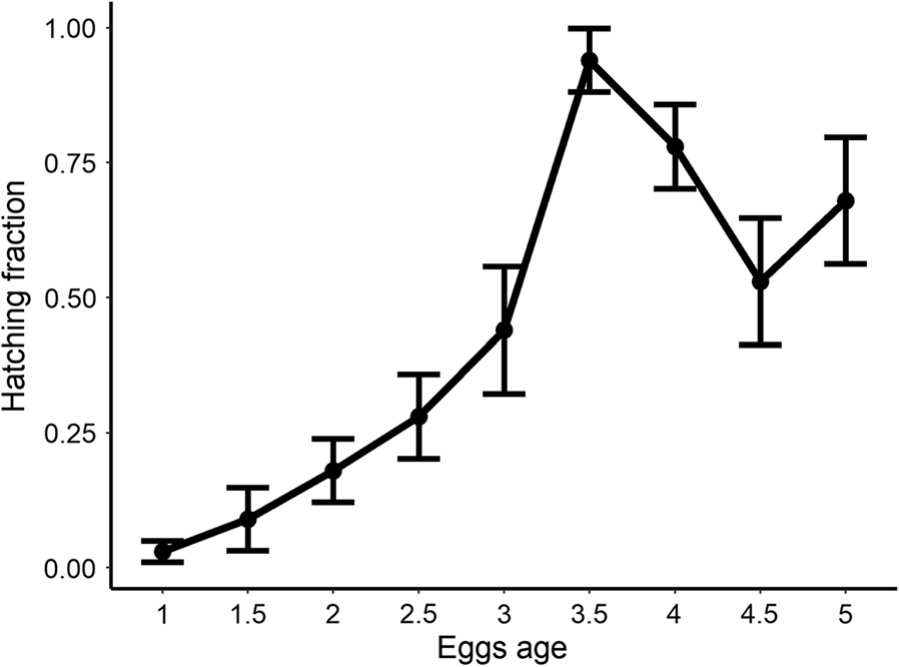
Fraction of hatched eggs for each tested age (in months). Points: average values. Vertical lines: 95% Confidence Intervals (average ± 1.96∙standard error, SE)

**Table 2:** Estimates, standard errors, and *P*-values of the parameters of the GLM associating hatching rates and age (in months)

Parameter	Coefficient estimate	Standard error	*z* value	*P*-value
Intercept	− 3.68	0.22	− 16.63	< 0.001
Age	1.09	0.07	15.36	< 0.001

Hatching rates are significantly different between different ages (Binomial tests’ *P*-values < 0.05) with the exception of the fractions observed for 3 and 4.5 months and 4 and 5 months.

Our findings highlight that *Ae. koreicus* diapausing eggs require at least 3 months in order to hatch with substantial success. The optimal age (0.94 probability of successful hatch) seems to be around 3.5 months (about 100 days). This is in accord with previous studies carried out for *Ae. albopictus*. In particular, a period of 90–100 days was required to ensure diapause termination for laboratory experiments [15–17]. In another study aimed at quantifying diapause duration, the authors estimated the time needed for half of *Ae. albopictus* diapausing eggs to hatch to be about 110 days [18]. Similarly, in [14] the authors estimated the duration of diapause in *Ae. j. japonicus* to be at least 3 months.

Moreover, this study points out that water immersion alone did not lead to the hatching of eggs, while fully developed embryos were observed in these eggs after a bleaching treatment. Our investigation thus confirms effective diapause for *Ae. koreicus* in northern Italy.

Our experiments improve our current scarce knowledge of *Ae. koreicus* biology and developmental cycle, providing important insights on how this species overwinters in Europe. Our findings could help in designing future experiments more effectively and improving modeling efforts aimed at predicting the invasion dynamics of this species, and therefore the potential risk for human and animal health [19, 20].

## Data Availability

All data generated or analyzed during this study are included in this published article.
